# Arthritis Caused by MRSA CC398 in a Patient without Animal Contact, Japan

**DOI:** 10.3201/eid2604.190376

**Published:** 2020-04

**Authors:** Hidemasa Nakaminami, Yuji Hirai, Hirosuke Nishimura, Shunsuke Takadama, Norihisa Noguchi

**Affiliations:** Tokyo University of Pharmacy and Life Sciences, Tokyo, Japan (H. Nakaminami, S. Takadama, N. Noguchi);; Tokyo Medical University Hachioji Medical Center, Tokyo (Y. Hirai, H. Nishimura)

**Keywords:** livestock-associated methicillin-resistant Staphylococcus aureus, Panton-Valentine leukocidin, CC398, ST1232, MRSA, antimicrobial resistance, One Health, AMR, bacteria, arthritis, Japan

## Abstract

Clonal complex 398 methicillin-resistant *Staphylococcus aureus* (MRSA) is a typical lineage of livestock-associated MRSA. We report a case of intractable arthritis of the shoulder joint caused by a multidrug-resistant Panton-Valentine leukocidin–positive livestock-associated MRSA clonal complex 398 sequence type 1232 clone in a patient in Japan who had no animal contact.

In the past decade, methicillin-resistant *Staphylococcus aureus* (MRSA) has been detected in livestock, including swine, poultry, and veal calves (*1*,*2*). In general, the virulence of animal-derived livestock-associated (LA-MRSA) strains is considered to be lower than that of community-acquired MRSA lineages (*3*). However, LA-MRSA strains can effectively colonize and infect humans, with subsequent transmission in both community and hospital settings. Human colonization with LA-MRSA sequence type (ST) 398 was first recognized among swine farmers in France and the Netherlands in the early 2000s (*4*). According to Larsen et al., clonal complex (CC) 398 MRSA accounted for 21% of MRSA isolated from skin and soft tissue infections in Denmark during 2010–2015 (*5*). However, ST398 MRSA has not been isolated in patients in Japan. We report a case of intractable arthritis of the shoulder joint caused by a multidrug-resistant Panton-Valentine leukocidin (PVL)–positive LA-MRSA CC398 (ST1232) clone in a patient in Japan who had no animal contact.

We performed MRSA identification, staphylococcal cassette chromosome (SCC) *mec* typing, *spa* typing, multilocus sequence typing (MLST), MIC determination, and PCR assays for detecting virulence factors and antimicrobial resistance genes, as described previously (*1*,*6*). The study protocol was approved by the Tokyo University of Pharmacy and Life Sciences Ethics Committee (approval no. 12–09).

The patient, a 74-year-old man who lives in Tokyo, had received dialysis treatments 3 times a week since April 2018. He reported no overseas travel or animal contact. In September 2018, he felt pain in his right shoulder joint and was admitted to the Tokyo Medical University Hachioji Medical Center. At admission, his leukocyte count was 18,600 cells/μL and his C-reactive protein level was 7.0 mg/dL; we began treatment with cefazolin immediately (day 0). We isolated MRSA from venous blood and joint fluid, and we switched the antimicrobial agent to vancomycin on day 1. 

Molecular epidemiologic analysis showed that the MRSA THI2018-120 strain we isolated is classified into SCC*mec* type V and *spa* type t034. Moreover, MLST analysis revealed that the strain was ST1232, a single-locus variant of ST398 that belongs to CC398. When we determined antimicrobial susceptibilities, the THI2018-120 strain exhibited multidrug resistance to oxacillin, gentamicin, clarithromycin, clindamycin, and tetracycline (Table). We detected resistance genes for aminoglycoside (*aacA-aphD*) and tetracycline (*tet*[K]). However, we did not find known macrolide resistance genes, including *ermA*, *ermB*, *ermC*, *ermT, mphC*, and *msrA/B*, or clindamycin resistance genes *lnuA*, *lnuB*, *lnuC*, and *lnuD* (*1*,*6*). We conducted experiments to detect virulence factors, which detected the *lukS/F-PV* genes (Table). In addition, the THI2018-120 strain carried *clfA*, *clfB*, and *fnbA*, which are microbial surface components recognizing adhesive matrix molecules (*6*).

**Table:** Antimicrobial drug resistance for clonal complex 398 sequence type 1232 staphylococcal cassette chromosome *mec* type V methicillin-resistant *Staphylococcus aureus* strain isolated from a patient in Japan*

Antimicrobial drug	MIC, μg/mL	Susceptibility
Ampicillin	4	ND
Oxacillin	8	R
Fosfomycin	0.5	S
Gentamicin	32	R
Levofloxacin	0.25	S
Clarithromycin	64	R
Clindamycin	>256	R
Tetracycline	128	R
Vancomycin	1	S
Daptomycin	0.5	S
*Antimicrobial resistance genes included *mecA*, *aacA-aphD*, and *tet(K)*. Virulence factors included *lukS/F-PV*, *clfA*, *clfB*, and *fnbA*. ND, breakpoint is not defined by Clinical and Laboratory Standards Institute criteria; R, resistant; S, susceptible.		

On day 18, we performed surgical debridement. On day 29, the patient had a drug reaction to vancomycin, so we switched the antimicrobial agent to daptomycin. On day 80, we added oral rifampin to the patient’s regimen to treat prolonged chronic osteomyelitis. The patient’s symptoms improved, and we switched from daptomycin to oral levofloxacin on day 108. On day 119, the patient was discharged when we no longer detected MRSA in pus from drained and nonopen lesions.

Human infections with the PVL-positive ST1232 MRSA strain are rare but were reported in New Zealand during 2011–2013 (*1*). In 2015, a fatal infection caused by a PVL-positive ST398 MRSA was reported in a patient who was infected in China but developed symptoms in Japan (*7*). 

As mentioned, the virulence of animal-derived ST398 MRSA strains is considered to be lower than that of community-acquired MRSA (*3*). However, we presume that PVL production enhanced the severity of this case. Recent surveillance data suggest that not all cases of MRSA CC398 occurring among humans are related to animals (*8*). Our patient had no connection to animals, which suggests that this strain might be more common in Japan than previously thought. Further investigation, including whole-genome analysis of this isolate, could provide accurate phylogeny with higher resolution. In addition, a more robust estimation of this strain’s virulence might elucidate the actual transmission route in this patient.

We previously reported the increased prevalence of the PVL-positive USA300 and USA300-LV clones in Japan, which were disseminated from North and Latin America (*6*). We also reported a case of septic arthritis by a PVL-positive ST772 Bengal-Bay clone, which is a predominant clone in India (*9*). Those data suggest that diverse and highly pathogenic PVL-positive MRSA clones have been entering Japan from abroad. We hypothesize that the PVL-positive ST1232 MRSA strain in this case also was transmitted from abroad. The transmission route of antimicrobial-resistant strains might be not only by humans but also by imported edible meat (*10*). From a One Health perspective, increased monitoring of imported livestock products is needed to prevent antimicrobial-resistant strains entering from other countries.
